# Regarding “Challenges in diagnosing thrombotic thrombocytopenic purpura”

**DOI:** 10.1016/j.htct.2025.103986

**Published:** 2025-09-20

**Authors:** Cilomar Martins de Oliveira Filho, Brian J. Carney

**Affiliations:** Beth Israel Deaconess Medical Center

*Dear Editor,*

We appreciate the thoughtful critique of our recent case report reported as Images in Clinical Hematology [1] and would like to clarify points raised regarding the diagnostic criteria for immune thrombotic thrombocytopenic purpura (iTTP).

In their response, Jacobs et al. [2] express concern that our case lacked sufficient information to confirm a diagnosis of iTTP, specifically citing the absence of reported ADAMTS13 autoantibody data and genetic testing. We agree with the importance of distinguishing immune-mediated from congenital TTP, as this has significant implications for management and prognosis.

The critique reads as follows:

*“As such, without the identification of an autoantibody, genetic testing should be performed to exclude mutations in the ADAMTS13 gene. Given that the authors did not report an antibody, nor did they assess for genetic mutations, this case cannot be considered a ‘confirmed’ case of iTTP.”*

We would like to clarify that an ADAMTS13 inhibitor screen was indeed performed in our patient and yielded a positive result**.** The inhibitor titer, measured using the Bethesda assay, was 2.2 (reference <0.4)**,** indicating the presence of a circulating autoantibody against ADAMTS13. This supports the diagnosis of acquired, immune-mediated TTP. Due to word count limitations and the case vignette format, this detail was not included in the original publication.

We agree that in the absence of detectable autoantibodies, the possibility of congenital TTP should be considered, and genetic testing may be warranted. However, in our case, the presence of a quantifiable inhibitor supports an immune-mediated process, and the clinical picture (including age of onset and concurrent autoimmune disease) makes congenital TTP unlikely.

We appreciate the ongoing discussion regarding the importance of diagnostic precision in TTP. We fully agree that thorough documentation of relevant laboratory findings is critical, not only for accurate diagnosis, but also for management and epidemiological purposes.

## Conflicts of interest

No conflicts of interest to declare.
